# An emotion-differentiated perspective on empathy with the emotion specific empathy questionnaire

**DOI:** 10.3389/fpsyg.2014.00653

**Published:** 2014-07-01

**Authors:** Sally Olderbak, Claudia Sassenrath, Johannes Keller, Oliver Wilhelm

**Affiliations:** ^1^Differentielle Psychologie und Psychologische Diagnostik, Institut für Psychologie und Pädagogik, Universität UlmUlm, Germany; ^2^Sozialpsychologie, Institut für Psychologie und Pädagogik, Universität UlmUlm, Germany; ^3^Knowledge Media Research CenterTübingen, Germany

**Keywords:** affective empathy, cognitive empathy, emotion, psychometric analysis, measurement model

## Abstract

Empathy refers to the thoughts and feelings of one individual in response to the observed (emotional) experiences of another individual. Empathy, however, can occur toward persons experiencing a variety of emotions, raising the question of whether or not empathy can be emotion specific. This paper discusses theoretical and empirical support for the emotion specificity of empathy. We present a new measure, the Emotion Specific Empathy questionnaire, which assesses affective and cognitive empathy for the six basic emotions. This paper presents the measure's psychometric qualities and demonstrates, through a series of models, the discriminant validity between emotion specific empathies suggesting empathy is emotion specific. Results and implications are discussed.

## Introduction

To effectively navigate the social world, it is important to understand others, infer their thoughts and feelings, and to affectively connect to their emotional experiences. Put differently, the extent to which one can empathize with others is a key component of a successful social interaction. Literature on empathy supports its strong role in effective social functioning: Empathy is related to various prosocial behaviors such as helping (e.g., Batson et al., 1989, 1991, 1997) and cooperation (e.g., Eisenberg and Miller, 1987). Patients interacting with empathic doctors recover more quickly (e.g., Van Dulmen and Bensing, 2002) and empathy improves intergroup relations (e.g., Dovidio et al., 2010). Empathy, however, can occur toward persons experiencing a variety of emotions, raising the question of whether or not empathy can be emotion specific.

This paper discusses theoretical and empirical support for the possible emotion specificity of empathy. In lieu of that research, we find that present measures of empathy are biased in that they do not take an emotion differentiated perspective on empathy. We present a possible solution to this problem by introducing a new measure, the Emotion Specific Empathy questionnaire, which was designed to assess emotion specific affective and cognitive empathy. We will present that measure, its psychometric qualities, and tests that suggest there may be emotion specific empathic reactions.

### Empathy

At large, empathy refers to the thoughts and feelings of one individual in response to the observed (emotional) experiences of another individual (e.g., Davis, 1983; cf. Woltin et al., 2011). However, similar to most psychological constructs, the specific definition of empathy is debatable. Batson (2009) identifies eight definitions of empathy that are currently in use; of these eight, we will expand on two that are commonly applied. The first definition refers to knowing the internal state of another person and has been referred to as cognitive empathy, perspective taking, empathic accuracy, and theory of mind (Batson's Concept 1). This type of empathy, referred to in this paper as *Cognitive Empathy*, is related to better negotiation outcomes and satisfaction with the negotiation process (e.g., Bazerman and Neale, 1982; Galinsky et al., 2008), reduced stereotypes (e.g., Todd et al., 2011), and enhanced intergroup contact (e.g., Falk and Johnson, 1977). The second definition proposed by Batson (2009) refers to feeling the “same emotion that another person feels” (page 5) and has been referred to as emotional contagion, affective empathy, and emotional empathy (Batson's Concept 3). This type of empathy, referred to in this paper as *Affective Empathy*, refers to an affective connection with another person's emotional state, and closely relates to an other-oriented reaction regarding the perceived welfare of the other person (cf. Batson et al., 1997). Affective empathy is related to behavioral mimicry (Dimberg et al., 2011) and the motor neuron system facilitating behavioral mimicry (Nummenmaa et al., 2008).

Although both types of empathy imply the processing of others' emotional states, emotion-specificity in both the concept as well as the assessment of cognitive and affective empathy has not yet been considered. In the following we will elaborate on why considering emotion specificity in empathy is worthwhile as it presents an advancement in the theorizing and measurement of the concept of empathy.

### Emotion specific empathy

In the following section, we present several arguments that suggest researchers may need to consider empathy from an emotion specific perspective. First, we refer to a prominent theoretical model of empathy, the Perception-Action Model (PAM) of empathy (cf. Preston and de Waal, 2002; Preston and Stansfield, 2008; Preston and Hofelich, 2012), which (implicitly) assumes emotion specific empathy. PAM assumes that for an empathic reaction to occur, the observer (i.e., the subject) must attend to another individual's (i.e., the object's) emotional state. By attending to the object's state, the subject's neural representations of similar states are automatically activated, thus, other-oriented object-congruent reactions are facilitated. Herein, PAM provides a theoretical framework that relates empathy to concepts such as facial mimicry (e.g., Sonnby-Borgström et al., 2003), emotional contagion (e.g., Blairy et al., 1999), and recognizing emotions in the face (e.g., Dimberg et al., 2011). PAM suggests that highly empathic individuals are those who are more successful and/or more motivated to attend to others' emotional states and thereby often have similar neural representations consequentially activated. This explains why those who are highly empathic show facial mimicry at an earlier stage of information processing (Sonnby-Borgström et al., 2003), are better at detecting others' emotions (Dimberg et al., 2011), and display more emotional contagion (Blairy et al., 1999).

Importantly, the entire process outlined in PAM implies emotion specificity given that the type of emotional state observed in the object by the subject should elicit different neural representations, setting the stage for different empathic responses and consequently leading to different facial mimicry reactions, emotional contagion, and behavioral reactions. Whereas most research has identified the same areas may be implicated for several emotions (cf. Cunningham et al., 2008; Kober et al., 2008; Shackman et al., 2011; Lindquist et al., 2012; Oosterwijk et al., 2012) some findings indicate that feeling different emotions are associated with activity in different brain regions (e.g., Lane et al., 1997; Pelletier et al., 2003; Tettamanti et al., 2012). However, the potential differentiation in brain region and neural networks, even at the level of positive and negative affect, suggests this differentiation might carry through to the level of specific emotions.

Nevertheless, the extent to which one can empathize with another individual may vary depending on the empathized emotion as the PAM suggests. Correspondingly, in other avenues, research suggests emotion specificity may exist in empathy related abilities, such as mimicry, emotion memory, and emotion recognition (e.g., Oberman et al., 2007; Ponari et al., 2012; Wilhelm et al., in press). An emotion specific conceptualization and measurement of empathy will instigate future research that disentangles the relation between, for example, empathizing with someone's sadness and mimicking his/her sadness, happiness, or fear. An emotion specific perspective on empathy appears more appropriate to us, not only because—as elaborated above—empathic reactions to sadness should imply different processes compared to empathic reactions to joy, anger, or disgust (cf. Lane et al., 1997; Preston and de Waal, 2002; Pelletier et al., 2003; Tettamanti et al., 2012), but also because being able to empathize with another's sadness (in contrast to another emotion such as anger) should be related to different behavioral outcomes and help us further understand individual differences in empathy.

Finally, on a behavioral level, we argue individuals will produce behavioral reactions that differ depending on the emotion of interest. For example, perceiving sadness in others increases affiliative tendencies and helping behavior while this is not the case for perceiving anger (Hendriks and Vingerhoets, 2006; Van Doorn et al., 2012). While affectively connecting (i.e., showing affective empathy) with a sad person increases one's willingness to help that person (see Batson et al., 1989, 1991, 1997), it is debatable whether affectively connecting with an angry person results in the same increased willingness to help. This is because anger and sadness differ in their underlying appraisals (cf. Frijda, 1986), which in turn affects behavioral consequences of these emotions (cf. Horstmann, 2003; Seidel et al., 2010). Anger, for example, is an emotion that arises when a sudden obstacle appears in the pursuit of one's goals, leading the subject to perceive unfairness and wish to fight the obstacle (Haidt, 2003). Consequentially, anger is associated with approach-related, aggressive behavioral tendencies (e.g., Carver and Harmon-Jones, 2009). Hence, it appears plausible that an individual who is sensitive to others' anger (i.e., a highly anger-empathic individual) might be more likely to engage in aggressive behavioral acts than if he or she was highly sadness- or fear-empathic.

Taken together, we argue that at least three main arguments speak to the fact that a differentiated perspective on empathy is well indicated. First, theoretical models of empathy suggest emotion specificity (cf. Preston and de Waal, 2002). Second, considering empathy as emotion specific yields new research questions and potentially important insights on the relation of empathy and related concepts and abilities such as mimicry, emotion recognition, and emotion memory, because different emotions are associated with different appraisals and information processing (cf. Frijda, 1986). Finally, given specific appraisals are associated with different emotions, different consequences might be associated with empathy when considered emotion specific.

### Emotion specific empathy questionnaire

Building on the rationale elaborated above, we decided to test whether or not individuals report differing levels of empathy depending on the type of emotion addressed. It is notable that established questionnaires assessing empathy, both on a cognitive as well as on an affective dimension, are geared mainly toward assessing reactions to the sadness causing misfortunes experienced by others. For example, the Empathic Concern subscale of the Interpersonal Reactivity Index (Davis, 1980), generally considered to assess affective empathy, contains items such as “I often have tender, concerned feelings for people less fortunate than me.” or “When I see someone being taken advantage of, I feel kind of protective toward them.” Likewise, Mehrabian and Epstein's (1972) measure of emotional empathy contains items such as “I cannot continue to feel OK if people around me are depressed.” or “Seeing people cry upsets me.” We argue that these items reflect the extent to which one can empathize with somebody in terms of compassion or pity.

Since current measures of empathy are lacking the consideration of emotion specificity in empathic reactions, we developed a new emotion specific measure of empathy called the Emotion Specific Empathy questionnaire. As a first approach to the topic, we decided to include, of all possible and plausible emotional reactions to others' emotional experiences, the six basic emotions (cf. Frijda, 1986; Ekman, 1992). There is some support for the existence of these emotion categories, in particular regarding the perception of facial emotion expressions (e.g., Elfenbein and Ambady, 2002), however the discreetness of these emotions is controversial with others proposing alternative structures to emotion (e.g., see the series of articles between Barrett, Panksepp, and Izard: Barrett, 2006; Barrett et al., 2007; Izard, 2007a,b; Panksepp, 2007). The development of the ESE with its base in the six basic emotions was meant as an initial approach to examine potential emotion or affect specific bias in empathy and is not meant to identify the only ways empathy might be emotion specific or exclude other ways empathy might be emotion specific. To do this, we designed a questionnaire that assesses individuals' capability to connect cognitively and affectively with others experiencing anger, disgust, fear, happiness, sadness, or surprise. We developed 12 subscales to assess specific affective or cognitive empathy for each of the six basic emotions. For example, the Anger Affective Empathy subscale assesses the extent to which one affectively connects with and feels another person's anger and the Anger Cognitive Empathy subscale assesses the extent to which one can cognitively take another person's perspective and understand why that person is angry.

We wrote 10 items, five specific to assessing affective empathy and five specific to assessing cognitive empathy, that were inspired by related empathy questionnaires, and included terms related to one of the six basic emotions. This was done for all six emotions, thus resulting in subscales that differed only in the emotional content they addressed. Then, through a series of analyses, we can test the convergent and discriminant validity of these scales.

The goal of this paper is to present a new empathy measure that assesses emotion specific affective and cognitive empathy. We will present the psychometric properties of that measure, including item and scale-level properties. Then, through a series of models, we will test for discriminant validity between emotion specificity in affective and cognitive empathy. Finally, we will compare our measure with an existing empathy measure.

## Methods

### Sample

Participants were recruited through the online Mechanical Turk survey website and were provided $8.00 for participating. Recent studies suggest Mechanical Turk can be used to obtain data from a demographically diverse sample with data that is comparable to samples collected via traditional methods (Buhrmester et al., 2011; Casler et al., 2013). In addition, research suggests that Mechanical Turk participants are similar to student samples on many personality characteristics, with the exception of extraversion and self-esteem (Goodman et al., 2013). Because in online testing participants are anonymous, one potential drawback of this methodology is participants will not complete the measures seriously. To control for this, we included two attention-check questions (recommended for Mechanical Turk studies; Goodman et al., 2013) and removed participants who showed no variance in their responses suggesting the person consistently selected the same response.

Initially, 616 individuals participated; we removed 127 participants because they incorrectly responded to the attention-check questions, and an additional three were removed because there was no variance in their response across an entire scale. Our final sample consisted of 486 participants (243 Women), primarily White, non-Hispanic (76%; 7% Black, non-Hispanic; 8% Asian; 9% Other), from a range of education levels (13% High School or GED equivalent, 33% Some College, 11% Associates Degree, 35% Bachelors Degree, 8% More than a Bachelors Degree).

### Materials and procedure

The Emotion Specific Empathy questionnaire was administered in a large online questionnaire study including other measures not discussed in this paper that took roughly 50 min to complete.

#### Emotion specific empathy (ESE)

The ESE consists of 60-items; five items per subscale (see **Table 2** for items^1^ organized by subscale and instructions for reverse coding). Participants are asked to “Please indicate how strongly you agree or disagree with the following statements. Use the scale below and write your answers in the spaces provided.” The measure uses a 7-point likert scale: -3, Disagree Strongly; -2, Disagree Somewhat; -1, Disagree Slightly; 0, Neutral; 1, Agree Slightly; 2, Agree Somewhat; 3, Agree Strongly. Scores for subscales are computed by taking an average across all relevant items.

#### Interpersonal reactivity index (IRI; Davis, 1980)

The IRI is a 28-item measure with a 5-point likert scale that assesses empathy through four subscales. In this study, we present results related to two of those subscales: (1) Empathic Concern, considered to measure affective empathy (α = 0.70); and Perspective Taking, considered to measure cognitive empathy (α = 0.88).

## Results

### Internal consistency

First, we evaluated the internal consistency of the ESE subscales. According to Cronbach's alpha (α, 1951), which estimates the average inter-item correlation, and Omega Total (ω, McDonald, 1999), which estimates the extent to which scale variance is due to a single general factor, each ESE subscale had adequate internal consistency (see Table 1 for estimates).

**Table 1:** **ESE Subscale Statistics**.

	**Anger Affective**	**Anger Cognitive**	**Disgust Affective**	**Disgust Cognitive**	**Fear Affective**	**Fear Cognitive**	**Happy Affective**	**Happy Cognitive**	**Sad Affective**	**Sad Cognitive**	**Surprise Affective**	**Surprise Cognitive**
α	0.87	0.75	0.91	0.76	0.89	0.74	0.89	0.85	0.90	0.81	0.89	0.78
ω	0.88	0.78	0.92	0.78	0.89	0.74	0.90	0.86	0.91	0.82	0.89	0.79
**COMPARING MEANS BETWEEN MEN AND WOMEN**												
**Men**												
Mean	4.11	5.36	3.96	5.09	3.93	5.17	5.16	5.74	4.46	5.53	3.97	5.16
*SD*	1.19	0.91	1.22	0.99	1.17	0.90	1.11	0.92	1.26	0.94	1.15	0.89
**Women**												
Mean	4.43	5.48	4.51	5.27	4.63	5.40	5.68	6.00	5.17	5.83	4.41	5.30
SD	1.23	0.89	1.26	0.91	1.25	0.88	1.01	0.84	1.14	0.81	1.20	0.89
*T*-test [*t*_(483)_]	2.94^*^	1.52	4.87^*^	2.10^*^	6.36^*^	2.83^*^	5.35^*^	3.31^*^	6.52^*^	3.75^*^	4.20^*^	1.72
Cohen's *d*	0.26	0.13	0.44	0.19	0.58	0.26	0.49	0.30	0.59	0.34	0.37	0.16
**CONFIRMATORY FACTOR ANALYSIS**												
**Original structure**												
χ^2^	50.81^*^	9.67	44.39^*^	39.51^*^	38.93^*^	65.26^*^	5.77	49.35^*^	46.62^*^	48.64^*^	43.39^*^	104.96^*^
χ^2^ *df*	5	5	5	5	5	5	5	5	5	5	5	5
SRMR	0.034	0.022	0.022	0.048	0.036	0.061	0.010	0.040	0.030	0.042	0.030	0.073
RMSEA	0.137	0.044	0.128	0.119	0.118	0.158	0.018	0.135	0.131	0.134	0.126	0.203
CFI	0.967	0.992	0.977	0.947	0.976	0.885	1.000	0.957	0.974	0.946	0.972	0.859
TLI	0.933	0.984	0.955	0.894	0.952	0.769	0.999	0.914	0.948	0.892	0.944	0.717
**With added covariances**												
χ^2^	5.39	na	13.11^*^	10.15^*^	10.53^*^	10.54^*^	na	8.68	9.66^*^	7.66	2.28	9.84^*^
χ^2^ *df*	3		4	3	4	4		4	4	4	4	4
SRMR	0.013		0.014	0.021	0.014	0.025		0.016	0.015	0.020	0.006	0.025
RMSEA	0.041		0.069	0.070	0.058	0.058		0.049	0.054	0.043	0.000	0.055
CFI	0.998		0.995	0.989	0.995	0.988		0.995	0.996	0.996	1.000	0.992
TLI	0.994		0.987	0.963	0.988	0.969		0.989	0.991	0.989	1.003^2^	0.979

* *p < 0.05; na = not applicable*.

The internal consistency of each subscale was also assessed by modeling each subscale in Confirmatory Factor Analysis (CFA) with Maximum Likelihood estimation and with each of the five subscale items predicted by a single latent construct. We chose to model the subscales individually, instead of modeling all 12 subscales in a single model, because a single model with all 60 items would have required us to estimate more parameters than is suggested with our current sample size. A ratio of 5:1, or 5 participants for every parameter, is considered to be the minimum ratio for a CFA (Kline, 2005) so with 486 participants, we should estimate no more than 97 parameters, which is less than the minimum of 120 parameters we would need for the 60 items, ignoring any covariances between the latent factors. Also, our goal was to identify model misfit for the individual subscales, which is harder to identify when the subscales are combined into a single model.

The subscale measurement models were evaluated according to the χ^2^ goodness-of-fit statistic, absolute fit indices [root mean square error of approximation (RMSEA; Steiger and Lind, 1980) and the standardized root mean squared residual (SRMR; Bentler, 1995)], and incremental fit indices [Comparative Fit Index (CFI; Bentler, 1990) and the Tucker-Lewis Index (TLI; Tucker and Lewis, 1973)]. Hu and Bentler (1999) recommended these fit indices, among others, in evaluating model fit and in particular the combination of SRMR with the RMSEA, CFI, and TLI. According to Hu and Bentler, the following values indicate the model is a good fit to the data: SRMR < 0.06, RMSEA < 0.06, CFI ≥ 0.95, and TLI ≥ 0.95.

There was some disagreement between the fit indices: Only two models showed poor fit to the data according to all of the fit indices, however eight of the remaining 10 models showed poor fit according to at least one fit index. Ten models were rejected according to the χ^2^ goodness-of-fit statistic and the RMSEA; however, the χ^2^ goodness-of-fit statistic is easily inflated by large samples, leading to a high likelihood the model will be rejected (Kline, 2005), and some suggest the RMSEA statistic is artificially inflated in models with low degrees of freedom (see Kenny et al., 2014), which is also a characteristic of these models. Eight models showed poor fit to the data according to SRMR, CFI, and/or TLI.

Next, we examined individually each of the 10 models that showed poor fit to the data according to one or more fit index for sources of model misfit; then, we compared these sources across subscales to identify any potential patterns in subscales. The modification indices for each of these 10 models suggested model fit could be improved with the addition of one to two covariances between residuals. We chose to model additional covariances, instead of removing items, in order to keep the item structure within each subscale similar to the others to help with the later evaluation of convergent and discriminant validity between the subscales. The added covariances ultimately reflected weak to moderate relations between items and, as will be discussed next, indicated no consistent pattern across subscales (see Table 2 for added covariances).

**Table 2:** **ESE items and CFA coefficients**.

**ESE subscales**	**CFA β**
**ANGER AFFECTIVE EMPATHY**	
(1) I am not easily infected by the anger of other people. (-)	0.484
(20) I feel angry when I see that something is happening to a stranger that makes him/her feel angry.	0.728
(22) When I see that my friend is angry about something, I easily feel angry as well.	0.767
(38) If a friend told me about an event in his/her life that made him/her feel angry, I will easily feel angry as well.	0.821
(39) I easily feel angry when the people around me feel angry.	0.909
Added covariances: Items 20 and 22 *r* = 0.07, Items 22 and 38 *r* = 0.17	
**ANGER COGNITIVE EMPATHY**	
(18) I have a hard time predicting what situations will make other persons angry. (-)	0.461
(25) I can easily think about events that will make my friends angry.	0.562
(31) If someone tells me about an event that made him/her angry, I can easily understand why that event made him/her angry.	0.754
(51) It is difficult for me to understand what makes my friends angry. (-)	0.676
(55) It is easy for me to understand why others become angry when something awful happens to them.	0.730
Added covariances: None	
**DISGUST AFFECTIVE EMPATHY**	
(10) If a friend told me about an event in his/her life that made him/her feel disgust, I will easily feel disgusted as well.	0.813
(23) When I see that my friend is disgusted about something, I easily feel disgust as well.	0.911
(24) I feel disgust when I see that something is happening to a stranger that makes him/her feel disgust.	0.889
(29) I am not easily infected by the disgust of other people. (-)	0.705
(59) I easily feel disgust when the people around me feel disgust.	0.844
Added covariances: Items 10 and 24 *r* = −0.10	
**DISGUST COGNITIVE EMPATHY**	
(5) It is difficult for me to understand what makes my friends disgusted. (-)	0.566
(8) If someone tells me about an event that made him/her feel disgusted, I can easily understand why that event made him/her disgusted.	0.803
(14) I can easily think about events that will make my friends disgusted.	0.418
(37) It is easy for me to understand why others become disgusted when something awful happens to them.	0.792
(49) I have a hard time predicting what situations will make other persons disgusted. (-)	0.580
Added covariances: Items 5 & 14 *r* = −0.12, Items 14 & 49 *r* = 0.15	
**FEAR AFFECTIVE EMPATHY**	
(4) I feel scared when I see that something is happening to a stranger that makes him/her feel scared.	0.656
(6) I am not easily infected by the fear of other people. (-)	0.611
(33) I easily feel scared when the people around me feel scared.	0.856
(45) If a friend told me about an event in his/her life that made him/her feel scared, I will easily feel scared as well.	0.856
(56) When I see that my friend is scared about something, I easily feel scared as well.	0.892
Added covariances: Items 4 and 6 *r* = 0.15	
**FEAR COGNITIVE EMPATHY**	
(3) It is easy for me to understand why others become scared when something frightening happens to them.	0.401
(11) I can easily think about events that will make my friends scared.	0.514
(26) I have a hard time predicting what situations will make other persons scared. (-)	0.691
(30) It is difficult for me to understand what makes my friends scared. (-)	0.726
(60) If someone tells me about an event that made him/her scared, I can easily understand why that event made him/her scared.	0.567
Added covariances: Items 3 and 60 *r* = 0.28	
**HAPPY AFFECTIVE EMPATHY**	
(28) I easily feel happy when the people around me feel happy.	0.855
(32) If a friend told me about an event in his/her life that made him/her feel happy, I will easily feel happy as well.	0.854
(34) I feel happy when I see that something is happening to a stranger that makes him/her feel happy.	0.731
(35) When I see that my friend is happy about something, I automatically feel happy as well.	0.892
(42) I am not easily infected by the happiness of other people. (-)	0.681
Added covariances: None	
**HAPPY COGNITIVE EMPATHY**	
(13) It is easy for me to understand why others become happy when something pleasant happens to them.	0.793
(17) It is difficult for me to understand what makes my friends happy. (-)	0.696
(41) I can easily think about events that will make my friends happy.	0.708
(46) I have a hard time predicting what situations will make other persons happy. (-)	0.638
(47) If someone tells me about an event that made him/her happy, I can easily understand why that event made him/her happy.	0.803
Added covariances: Items 41 and 46 *r* = 0.19	
**SAD AFFECTIVE EMPATHY**	
(2) I easily feel sad when the people around me feel sad.	0.862
(36) If a friend told me about an event in his/her life that made him/her feel sad, I will easily feel sad as well.	0.825
(44) I feel sad when I see that something is happening to a stranger that makes him/her feel sad.	0.838
(48) When I see that my friend is sad about something, I easily feel sad as well.	0.820
(54) I am not easily infected by the sadness of other people. (-)	0.672
Added covariances: Items 44 and 48 *r* = 0.12	
**SAD COGNITIVE EMPATHY**	
(12) It is easy for me to understand why others become sad when something heartbreaking happens to them.	0.696
(15) It is difficult for me to understand what makes my friends sad. (-)	0.725
(43) I can easily think about events that will make my friends sad.	0.533
(52) I have a hard time predicting what situations will make other persons sad. (-)	0.580
58) If someone tells me about an event that made him/her sad, I can easily understand why that event made him/her sad.	0.807
Added covariances: Items 43 and 52 *r* = 0.22	
**SURPRISE AFFECTIVE EMPATHY**	
(7) When I see that my friend is surprised about something, I easily feel surprise as well.	0.864
(9) I am not easily infected by the surprise of other people. (-)	0.738
(19) I easily feel surprise when the people around me feel surprise.	0.837
(40) If a friend told me about an event in his/her life that made him/her feel surprise, I will easily feel surprised as well.	0.757
(53) I feel surprise when I see that something is happening to a stranger that makes him/her feel surprise.	0.713
Added covariances: Items 40 and 53 *r* = 0.15	
**SURPRISE COGNITIVE EMPATHY**	
16) It is easy for me to understand why others become surprised when something unexpected happens to them.	0.440
(21) I have a hard time predicting what situations will make other persons surprised. (-)	0.664
(27) If someone tells me about an event that made him/her surprised, I can easily understand why that event made him/her surprised.	0.598
(50) It is difficult for me to understand what makes my friends surprised. (-)	0.730
(57) I can easily think about events that will make my friends surprised.	0.714
Added covariances: Items 16 and 27 *r* = 0.34	

*The numbers presented before each item indicate that item's order of presentation in the questionnaire. (-) indicates the item should be reverse coded when scored*.

The recommended additional covariances between the residuals of affective empathy subscale items were primarily between three item types: (1) “I feel *angry* when I see that something is happening to a stranger that makes him/her feel *angry*.” (2) “When I see that my friend is *angry* about something, I easily feel *angry* as well.” and (3) “If a friend told me about an event in his/her life that made him/her feel *angry*, I will easily feel *angry* as well.” (where the emotional word, italicized in these examples, changed depending on the emotion of interest). However, the covariances only occurred in one or two subscales for the same pair of items and were always weak in nature, suggesting there was not a consistent pattern among the item types across the affective empathy subscales. The recommended additional covariances between the residuals of cognitive empathy subscale items were not specific to any item. For three subscales (Disgust, Happy, and Sad Cognitive Empathy), there was a weak positive relation between “I can easily think about events that will make my friends *disgusted*” and “I have a hard time predicting what situations will make other people *disgusted*.” The two strongest added covariances were for the Fear and Surprise Cognitive Empathy subscales between item types “It is easy for me to understand why others become *scared* when something *frightening* happens to them.” and “If someone tells me about an event that made him/her *scared*, I can easily understand why that event made him/her *scared*.” Like with the affective subscale items, there was not a consistent pattern across all cognitive empathy subscales suggesting there was not a systemic problem in the structure of the affective or cognitive empathy items. In the final CFAs, with the added covariances, all items showed moderate to strong relations to the single construct (see Tables 1, 2). Overall, these results suggest the ESE subscales show sufficient internal consistency.

### Subscale statistics

Next, we computed the mean values of the subscales. On all subscales women self-reported higher scores compared with men, which is consistent with other self-report empathy scales (e.g., Jolliffe and Farrington, 2006; Carré et al., 2013). These differences were significant for all subscales, with the exception of Anger Cognitive Empathy and Surprise Cognitive Empathy, however the effect sizes, assessed with Cohen's *d* (Cohen, 1992), were all small to medium (see Table 1).

The mean values of the subscales were correlated with each other to test the convergent and discriminant validity between the subscales (see Table 3; Campbell and Fiske, 1959). The items were derived from the same stem by instantiating emotion specific content so any reduction in the correlation from 1.0 would indicate a discrimination between the subscales that could be attributable to the emotion content differences. The correlations between the ESE affective subscales range from moderately to strongly related (*r*'s range from 0.43 to 0.77; average *r* = 0.63; Cohen, 1992), with the average shared variance amongst the scales at 40%, suggesting that while the subscales are similar to one another they are not perfectly related and are instead distinct. Correlations between the cognitive subscales were stronger compared with the strength of the relations between the affective subscales (*r*'s range from 0.64 to 0.79; average *r* = 0.70; average *R*^2^ = 0.49); likewise, the strength of these correlations and the average shared variance suggest the emotion specific cognitive empathy subscales are also distinct. Finally, correlations between the emotion congruent subscales ranged from moderate to strongly related (*r*'s range from 0.35 to 0.60; average *r* = 0.45; average *R*^2^ = 0.20) suggesting that while distinct, there is convergence (indicated by moderate to strong relations between the subscales and an average shared variance above null) between the emotion congruent empathy subscales. These correlations were in general larger than the correlations between the affective and cognitive empathy subscales that were emotion incongruent (*r*'s range from 0.14 to 0.48; average *r* = 0.31; average *R*^2^ = 0.10) suggesting discriminant validity between the affective and cognitive empathy subscales and between emotions.

**Table 3:**
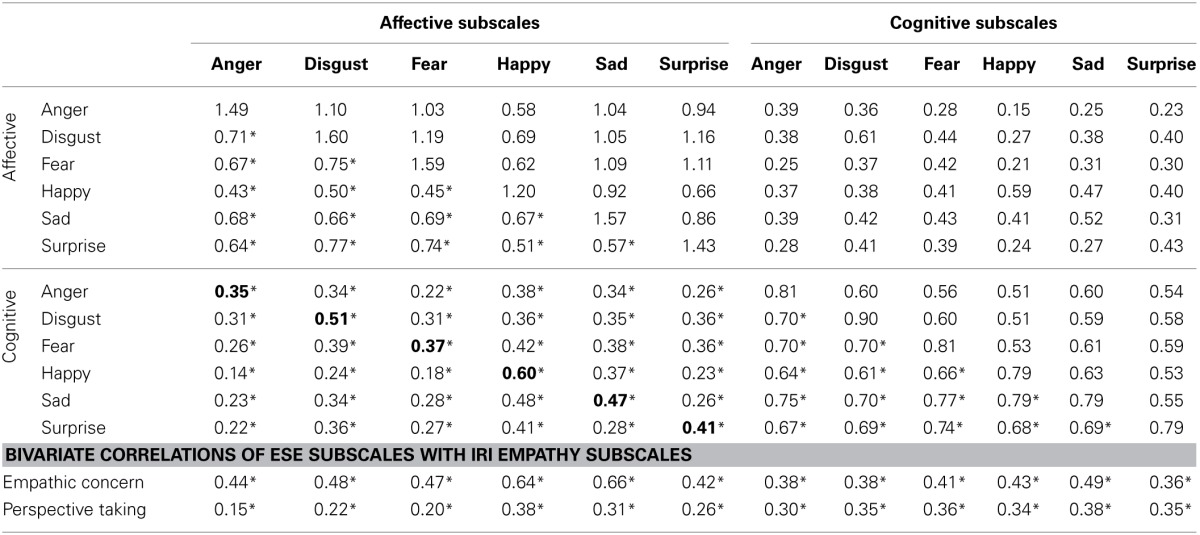
**ESE subscale correlations and covariances**.

^*^p < 0.05; In the top half of the table, correlations are presented below the diagonal, variances on the diagonal, and covariances above the diagonal. Correlation coefficients in the top left quadrant indicate the convergent validity between the affective empathy subscales and correlation coefficients in the bottom right quadrant indicate the convergent validity between the cognitive empathy subscales. Correlation coefficients in the bottom left quadrant indicate the discriminant validity between the emotion specific affective and cognitive empathy subscales, with the exception of the those coefficients presented in boldface, which indicate the convergent validity between the emotion specific subscales (i.e., Anger Affective Empathy with Anger Cognitive Empathy). The bottom half of the table presents correlations between the ESE and IRI subscales.

Finally, the ESE subscales were correlated with Empathic Concern and Perspective Taking, from the IRI. When correlated with Empathic Concern, all of the ESE affective subscales show stronger relations compared with their ESE cognitive empathy counterpart. And, when correlated with Perspective Taking, all of the ESE cognitive empathy subscales, with the exception of happy show stronger relations compared with their ESE affective empathy counterpart.

### ESE measurement model

**Figure 1:**
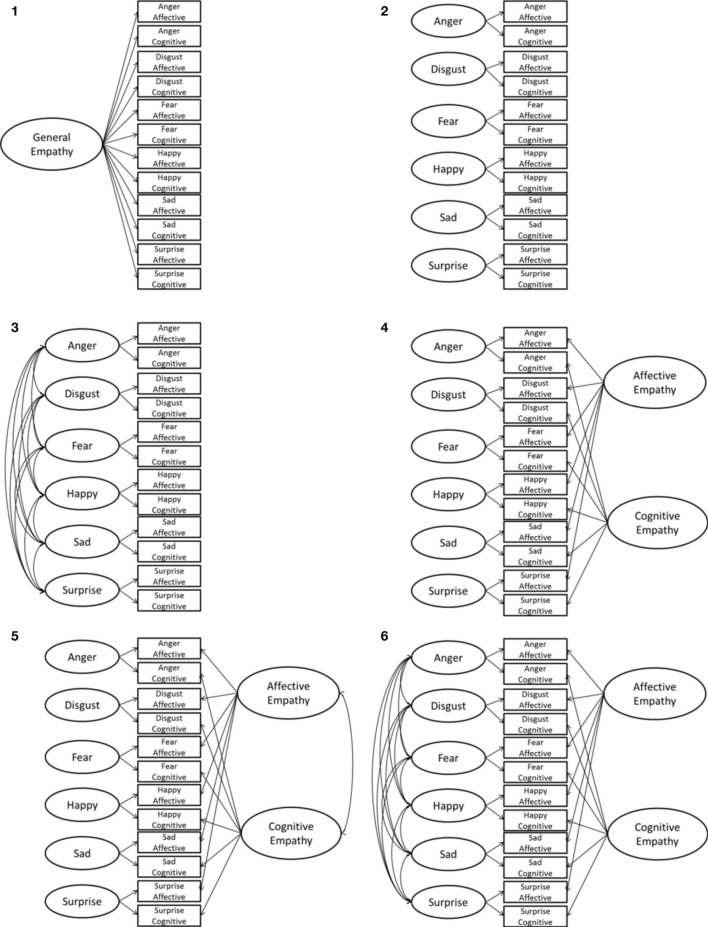
**Hypothesized Measurement Model Structures based on the MTMM framework**.

Because there is a lot of overlap between the subscales in terms of what they measure (i.e., affective subscales measure affective empathy, however each affective subscale should also measure an emotion specific empathy and thus is conceptually related to its emotion-specific cognitive empathy counterpart), we tested a series of hypothesized measurement model structures for the ESE measure, with the 12 subscales as indicators, that apply constraints to test specific hypotheses about the structure of the ESE measure and the differentiation between emotion specific empathies. Because we hypothesized emotion specific differentiation in empathy, the presented models focus on an emotion specific differentiation structure to the ESE measure. Each model tests specific hypotheses regarding the convergent and discriminate validity of the emotion specific empathies, ranging from complete discrimination between constructs to complete convergence. These models apply various constraints in a Multitrait-Multimethod framework (MTMM; Widaman, 1985; see Figure 1 for hypothesized models). Because of known convergence problems with the final solution of correlated trait and method factors, this structure was not modeled (Kenny and Kashy, 1992).

**Figure 2:**
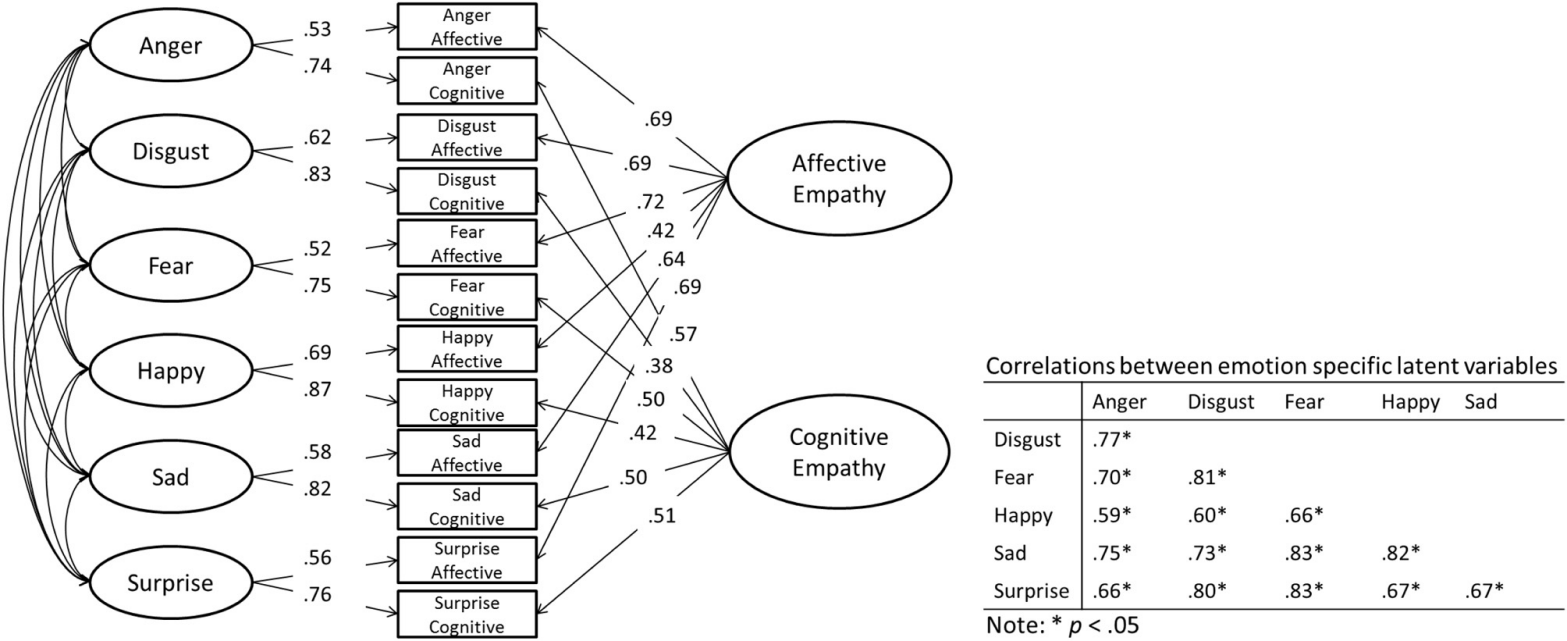
**Final ESE Measurement Model (Model 6)**.

Regarding the modeling of method variance, we opted for the inclusion of two methods factors (i.e., an affective empathy latent variable and a cognitive empathy latent variable) instead of the correlated uniqueness model (Marsh, 1989) or the correlated-trait correlated-method-1 structure (CTCM-1; Eid et al., 2008) in order to identify latent constructs for each empathy construct measured by ESE, so that later these latent constructs are available for testing covariate relations. In addition, the correlated uniqueness model is known to have bias when there are strong loadings on methods factors and strong correlations between method factors, which is a characteristic of our data (see Figure 2 below for loadings on method factors; Conway et al., 2004). Because the emotion factors are indicated by only two manifest variables, we added an additional constraint to adequately identify the emotion latent variable by constraining the unstandardized loadings from both manifest variables to equality forcing the affective and cognitive emotion subscale to load equally on the corresponding emotion latent factor^3^. Through an evaluation of model fit indices we can identify the best fitting structure to the data (see Table 4).

**Table 4:** **Measurement model fit**.

**Model**	**Model fit**
(1) One general empathy factor	χ^2^_(54)_ = 2182.85, *p* < 0.05, SRMR = 189, RMESA = 0.285_(0.275−0.295)_, CFI = 0.571, TLI = 0.476
(2) Orthogonal emotion factors	χ^2^_(60)_ = 4323.95, *p* < 0.05, SRMR = 0.461, RMESA = 0.383_(0.373−0.393)_, CFI = 0.141, TLI = 0.055
(3) Covarying emotion factors	Improper solution (predicted covariance matrix is not positive definite)
(4) Orthogonal emotion factors and orthogonal affective and cognitive factors	χ^2^_(48)_ = 430.79, *p* < 0.05, SRMR = 0.221, RMESA = 0.128_(0.117−0.140)_, CFI = 0.923, TLI = 0.894
(5) Orthogonal emotion factors and covarying affective and cognitive factors	χ^2^_(47)_ = 337.47, *p* < 0.05, SRMR = 0.073, RMESA = 0.113_(0.102−0.124)_, CFI = 0.942, TLI = 0.918
(6) Covarying emotion factors and orthogonal affective and cognitive factors	χ^2^_(33)_ = 147.24, *p* < 0.05, SRMR = 0.048, RMESA = 0.085_(0.071−0.099)_, CFI = 0.977, TLI = 0.954

First, we modeled a single general empathy factor for all 12 ESE subscales (Model 1), suggesting a single type of empathy was measured by all subscales; however, this model showed poor fit to the data. Next, we modeled emotion specific latent variables, which were either constrained to be orthogonal (Model 2) or allowed to covary (Model 3); both showed poor fit (with Model 3 producing an improper solution, most likely because the emotion factors are indicated by only two variables each, something that Gerbing and Anderson, 1985, 1987 found often results in models with improper solutions) suggesting modeling only differentiation between emotion specific empathies cannot account for the data.

The last three models (Models 4–6) included an affective and cognitive latent variable, indicated by the six affective or cognitive empathy scales, that were constrained to be either orthogonal to one another or covary. Of these three models, Model 6 showed acceptable fit, with the exception of the RMSEA statistic (which will be discussed next), and showed the best fit compared to the other five models, so this model was selected as our final measurement model^4^ (see Figure 2).

The RMSEA statistic for Model 6 was above the recommended value of 0.06 made by Hu and Bentler (1999); however, the value is also considered mediocre fit by MacCallum et al. (1996) and good fit by Steiger (1989). RMSEA can be easily inflated when a model has the correct number of specified factors but has incorrectly omitted a single covariance between residuals (Savalei, 2012). An examination of the modification indices suggests that the addition of a covariance between the residuals of Happy Affective Empathy and Sad Affective Empathy, which is weak in size (*r* = 0.12, *p* < 0.05), results in a lower RMSEA statistic [χ^2^_(32)_ = 97.50, *p* < 0.05, SRMR = 0.044, RMESA = 0.065_(0.051−0.080)_, CFI = 0.987, TLI = 0.973]. However, we refrain from including this covariance in our final measurement model because we did not specify this covariance a priori (Steiger, 1990), the additional covariance will most likely not replicate in future samples (MacCallum et al., 1992), and omitting this covariance will most likely not impact the basic correlational pattern between latent factors (Newcomb and Bentler, 1988).

Model 6 identified orthogonal affective and cognitive factors, which suggest the ESE measure has adequate differentiation between the affective and cognitive subscales, and emotion specific latent factors, suggesting there is emotion specific empathy for the six basic emotions. The emotion latent variables were allowed to correlate with one another, showing strong correlations with one another, suggesting that individuals who self-report high scores on one emotion empathy will also report high scores on the other empathies. This model also supports our earlier hypothesis of emotion specific differentiation in empathy.

### ESE and the IRI

Next, we compared the ESE with the Empathic Concern and Perspective Taking subscales of the IRI. The mean score for both IRI subscales were included as manifest variables and were correlated with the respective affective or cognitive latent variable as well of each of the emotion specific latent variables (see Table 5 for correlation coefficients)^5^. Empathic Concern was moderately related with Affective Empathy and Perspective Taking was moderately related with Cognitive Empathy respectively. Empathic Concern showed stronger relations with the emotion specific latent variables, compared with Perspective Taking, and interestingly, both Empathic Concern and Perspective Taking showed the strongest relation with Sad Empathy. These results support our earlier suggestion that the IRI seems to include a focus on sadness.

**Table 5:** **Correlation coefficients between the IRI subscales and the ESE latent variables**.

**ESE latent variable**	**Empathic concern**	**Perspective taking**
Affective empathy	0.29^*^	–
Cognitive empathy	–	0.31^*^
Anger empathy	0.49^*^	0.22^*^
Disgust empathy	0.40^*^	0.26^*^
Fear empathy	0.52^*^	0.30^*^
Happy empathy	0.58^*^	0.30^*^
Sad empathy	0.69^*^	0.35^*^
Surprise empathy	0.45^*^	0.31^*^

* *p < 0.05*.

## Discussion

### Summary

We developed and tested a questionnaire that assesses individual differences in self-reported emotion specific empathy behavior. The purpose of this paper was to identify if there is any emotion specific bias in empathic reactions. To test that research question, we designed a measure that presented six equivalent cognitive empathy subscales and six equivalent affective empathy subscales that differ only in the emotional term used. Our results suggest that while the affective empathy subscales are strongly correlated with one another, as well as the cognitive empathy subscales with each other, these correlations are far from unity, with the average shared variance between the subscales at 40% (affective empathy subscales) and 49% (cognitive empathy subscales). These results suggest that there is some emotion specificity in empathy, which of course may not be limited to these six emotions. Based on these results we suggest that the empathy construct is broader than is currently measured and that emotion specificity is something empathy researchers should consider.

The proposed new measure of emotion specific empathy shows adequate internal consistency (as is indicated by Cronbach's alpha, Omega, and the CFAs) and appropriate relations with external constructs (as is indicated by the correlations in Table 3). Based on the bivariate correlations, the ESE subscales are related yet distinct from one another, supporting our hypothesis that empathy is emotion specific and can be measured as such. The reported results also suggest that we can identify emotion specific empathy at both the level of general empathy, as evidenced by the emotion specific latent constructs modeled in the CFA, and the level of affective and cognitive empathy, as indicated by the correlations between the ESE subscales. When modeled in a measurement model, we see differentiation between the emotion specific empathies. Finally, we see that the IRI subscales, when correlated with the ESE affective, cognitive, and the emotion specific empathies, show the strongest relations with sadness empathy. These results suggest that these IRI subscales are selective in that they are particularly focused on sadness empathy.

### Limitations and future directions

The ESE measure is structured based on the six basic emotions (anger, disgust, fear, happy, sad, and surprise). This is just one framework through which to address emotion, however given the emotion bias we found, we suggest that future research should test other proposed emotion structures, such as examining positive and negative affect. While the ESE measure includes more negative affect subscales than positive, we attempted to test this structure with the current data. We remodeled the data and removed the emotion specific latent variables and instead modeled only two latent variables, one for negative empathy (indicated by the ESE items for anger, disgust, fear, and sad empathy) and one for positive empathy (indicated by the ESE items for happy empathy). Because we modeled these structures at the item level, the models were specific to only affective or only cognitive empathy, because otherwise with 50 manifest variables (i.e., 5 items per affective and cognitive empathy subscale), that model would have required us to estimate more parameters than is suggested with our current sample size. Modeling the data in this way presumes that there is only a positive and negative specific empathy, and that the emotion categories are not a proper structure to the data. However, model fit for both affective and cognitive empathy was poor^6^ suggesting that this structure did not fit the data. The measure, however, contains more items assessing emotions considered to have a negative valence, compared with the items considered to have a positive valence, which leads to perhaps a better identification of negative affect than the data allows for positive affect. A proper balance of positive and negative emotions would allow us a better dataset to properly test for higher-order positive and negative affective and/or cognitive empathy factors. This is fruitful direction for future research, however it was not addressed here.

### General discussion

The measure presented in this article, for the first time, differentiates cognitive and affective empathic responses for the six basic emotions (Frijda, 1986). In our view, the identification of an emotion specific bias in empathy represents an important new contribution to the conceptualization and measurement of empathy. As mentioned in the beginning, theoretical models of empathy imply in their assumptions that empathic responses should depend on the type of emotion that is empathized (cf. Preston and de Waal, 2002). In addition, research suggests empathy related concepts, including facial mimicry and recognition of emotions in the face, are emotion specific in their relations to each other (cf. Oberman et al., 2007; Ponari et al., 2012). Hence, assessing empathy in an emotion specific manner represents the consequential next step when studying empathy. As a result, we presented a new measure that differentiates empathic traits depending on emotion.

Measuring empathy emotion specifically may help solve observed empirical inconsistencies regarding empathy and empathy related constructs. For example, whereas some researchers document a relation between empathy and facial mimicry (cf. Sonnby-Borgström et al., 2003) others find no relation (e.g., Rives Bogart and Matsumoto, 2010). Possibly, these inconsistencies result from a too undifferentiated measurement of empathy. If emotion specific empathic responses exist, and this is what our data suggest, then there should be differences between individuals in their emotion specific empathy; for example, some being more anger- than sadness-empathic while others are more sadness- or happiness-empathic. Likewise, and as elaborated in the introduction, it is also plausible to assume that different types of emotion specific empathy relate to different levels of mimicking specific emotions. Highly sadness-empathic individuals might be “good” sadness mimickers but might not show enhanced mimicry regarding anger or happiness. In addition, it is likewise plausible to expect empathy related behavioral outcomes (e.g., helping behavior) to be moderated by emotion specificity. Whereas sadness empathy should be related to increased helping behavior (in fact, this is documented given that so far empathy has been conceptualized as an empathic response to other's sadness, cf. Mehrabian and Epstein, 1972; Davis, 1983; Batson et al., 1989, 1997), this is not necessarily the case for anger or disgust empathy.

As elaborated above, anger arises when individuals are confronted with sudden obstacles in the pursuit of their goal, leading to an urge to go against this obstacle (Haidt, 2003). More importantly, anger is associated with perceiving oneself possessing sufficient resources (i.e., coping potential) to deal with the impediment (e.g., to lash out; Roseman et al., 1994). Hence, relating with someone's anger should also be informative about the observer indicating the observer perceives him- or herself capable to deal with the situation (as this appraisal is essential for anger to be experienced; cf. Frijda, 1986). This in turn suggests that helping behavior as a consequence of anger empathy is not particularly likely to emerge and instead suggests the observer may actually react with aggressive behavior in an anger-empathic state.

In lieu of the possibility that the behavioral consequences of empathy differ depending on the empathized emotion, our understanding and conceptualization of empathy becomes more differentiated. So far, literature on empathy and its consequences suggests empathy is *the* competence for beneficial social interaction and communication; we referred to these benefits at the start of this paper (cf. Eisenberg and Miller, 1987; Batson et al., 1989, 1991, 1997; Dovidio et al., 2010). However, it might be worthwhile to consider the possibly detrimental consequences of empathy when conceptualized emotion specifically. As illustrated above, anger empathy might have different behavioral implications than sadness empathy. While fostering sadness empathy probably yields positive outcomes for social interactions, this might not be the case for anger empathy. These implications should be considered when implementing empathy-based trainings, for example, to reduce stereotypes or racial biases (cf. Batson et al., 1997; Dovidio et al., 2010).

Considering empathy as emotion specific opens up numerous new lines of research. Given the potential differences in behavioral reactions that differ as a result of felt emotion, next steps in research should test for behavioral correlates associated with emotion specific empathy; for example, testing differences in helping behavior or stereotype bias as they relates to emotion specific empathy. In addition, future research could use experimental studies for further validation of the emotion specificity of this construct; for example, testing for emotion specific differences in empathy related constructs such as facial mimicry.

Finally, we believe that the notion of emotion specific empathy also bears some applied value for empathy interventions that have become popular in a variety of work or clinical contexts (cf. Day et al., 2010). Empathy trainings should be designed according to a person's empathic traits, not on an overall level but rather in a differentiated manner, considering that feeling with someone experiencing something sad and being able to understand what thoughts are related to this experience does not necessary apply to the situation in which that person feels anger.

Taken together, given the prominent role of empathy in successful social communication and interaction, the present approach contributes to a better understanding of the concept of empathy itself but also of individual differences in this trait.

## Conflict of interest statement

The authors declare that the research was conducted in the absence of any commercial or financial relationships that could be construed as a potential conflict of interest.

## Footnotes

^1^We have also developed a German version of the ESE, which is available in the Supplementary Material.

^2^TLI can sometimes fall outside of the 0–1 range (Kline, 2005).

^3^Models 1–6 were also modeled with the unstandardized loadings on each emotion specific empathy model constrained so that they summed to one (cf. Little et al., 2007). However, model fit was always worse than the fit presented in Table 4, or resulted in models with improper solutions with the predicted covariance matrices not positive definite, so these results were not presented.

^4^To test that the model fit is not attributable to the orthogonal affective and cognitive empathy latent variables, we estimated a seventh model that included no emotion specific latent factors but instead only an affective empathy and a cognitive empathy latent factor that were orthogonal to one another. That model showed poor fit to the data, χ^2^_(54)_ = 953.31, *p* <0.05, SRMR = 238, RMESA = 0.185_(0.175–0.196)_, CFI = 0.819, TLI = 0.779.

^5^We chose to model a correlation between the Affective Empathy latent variable with Empathic Concern, and a correlation between Cognitive Empathy latent variable with Perspective Taking because those relations were theoretically specified, and we did not model a correlation between the Affective Empathy latent variable with Perspective Taking or Cognitive Empathy latent variable with Empathic Concern because those relations were not hypothesized beforehand. When modeled, it is apparent that the ESE Affective Empathy is not similar to Perspective Taking (*r* = −0.03) nor is the ESE Cognitive Empathy latent variable related to Empathic Concern (*r* = −0.57).

^6^Model fit for affective empathy was χ^2^_(274)_ = 2507.88, *p* < 0.05, SRMR = 077, RMESA = 0.130_(0.125−0.134)_, CFI = 0.775, TLI = 0.753, and for cognitive empathy the model fit was χ^2^_(274)_ = 2088.78, *p* < 0.05, SRMR = 085, RMESA = 0.117_(0.112−0.122)_, CFI = 0.739, TLI = 0.714.
