# Supplementary material for: An emotion-differentiated perspective on empathy with the emotion specific empathy questionnaire
Source: Front Psychol. 2014 Jul 1;5:653. doi: 10.3389/fpsyg.2014.00653 (PMC4076670; doi:10.3389/fpsyg.2014.00653)
Supplement: Supplementary file 1 [file DataSheet1.DOCX]

**Appendix A : German Version of the Emotion Specific Empathy questionnaire**

Die folgenden Aussagen erfragen Ihre Gedanken und Gefühle in verschiedenen Situationen. Bitte geben Sie bei jeder Aussage an, wie sehr Sie mit den folgenden Aussagen übereinstimmen. Zu diesem Zweck steht Ihnen eine Skala von "stimme gar nicht zu" (-3) bis "stimme vollkommen zu"(3) zu Verfügung. Bitte kreuzen Sie für Ihre Angabe die entsprechende Zahl an.

-3 -2 -1 0 +1 +2 +3

stimme gar stimme nicht zu vollkommen zu vollkommen zu

1. Ich werde auch leicht ärgerlich, wenn andere Leute sich über etwas ärgern.
2. Ich lasse mich nicht so leicht von der Trauer anderer Leute anstecken.
3. Ich kann es sehr gut nachvollziehen, warum andere Angst bekommen, wenn ihnen etwas Ängstigendes geschieht.
4. Ich ängstige mich leicht, wenn ich sehe, dass anderen etwas passiert was sie ängstigt.
5. Es fällt mir schwer, zu verstehen, was meine Freunde ekelt.
6. Ich lasse mich nicht so leicht von der Angst anderer Leute anstecken.
7. Wenn ich sehe, dass sich jemand erschreckt, kann es ein, dass ich mich mit erschrecke.
8. Wenn jemand mir von etwas erzählt, das ihn bzw. sie ekelt, kann ich leicht verstehen, warum es ihn/sie ekelt.
9. Ich lasse mich nicht so leicht von der Überraschung anderer Leute anstecken. Wenn andere Personen mir von etwas erzählen, was sie ekelt, kann ich das leicht nachempfinden.
10. Ich kann es leicht nachvollziehen, wenn meine Freunde sich ängstigen.
11. Ich kann es leicht nachvollziehen, wenn meine Freunde traurig sind.
12. Ich kann es sehr gut verstehen, warum Andere sich freuen, wenn ihnen etwas Angenehmes passiert.
13. Ich kann es leicht nachvollziehen, wenn meine Freunde sich ekeln.
14. Ich kann es sehr gut verstehen, warum andere traurig sind, wenn ihnen etwas Trauriges passiert.
15. Ich kann es sehr gut verstehen, warum andere überrascht sind, wenn ihnen etwas Unerwartetes passiert.
16. Ich kann es leicht nachvollziehen, wenn meine Freunde sich freuen.
17. Es fällt mir schwer, vorauszusehen, welche Situationen andere Personen ärgern.
18. Ich kann es leicht nachvollziehen, wenn meine Freunde überrascht sind.
19. Ich werde ärgerlich, wenn ich sehe, dass jemand anderem etwas passiert das ihn/sie ärgert.
20. Es fällt mir schwer, vorauszusehen, welche Situationen andere überraschen.
21. Wenn ich bemerke, dass eine andere Person sich über etwas ärgert, kann ich mich leicht mitärgern.
22. Wenn ich bemerke, dass andere Personen sich vor bestimmten Dingen ekeln, ekele ich mich auch leicht.
23. Ich ekele mich, wenn ich bemerke, dass jemand anderem etwas passiert, dass ihn/sie ekelt.
24. Ich kann es leicht nachvollziehen, wenn meine Freunde sich ärgern.
25. Es fällt mir schwer, vorauszusehen, welche Situationen Andere ängstigen.
26. Wenn jemand mir von etwas erzählt, das ihn bzw. sie überrascht, kann ich leicht verstehen, warum es ihn/sie überrascht.
27. Wenn ich sehe, dass ein Freund von mir sich über etwas freut, freue ich mich auch.
28. Ich lasse mich nicht so leicht vom Ekel anderer Leute anstecken.
29. Es fällt mir schwer zu verstehen, was meine Freunde ängstigt.
30. Wenn jemand mir von etwas erzählt, das ihn bzw. sie ärgert, kann ich leicht verstehen, warum es ihn/sie ärgert.
31. Ich kann mich sehr leicht mit anderen mitfreuen.
32. Ich ängstige mich auch leicht, wenn andere um mich herum Angst haben.
33. Wenn mir ein Freund/eine Freundin von etwas erzählt, das ihn bzw. sie freut, kann ich mich leicht mitfreuen.
34. Ich lasse mich nicht so leicht von der Freude anderer Leute anstecken.
35. Ich fühle mich leicht traurig, wenn andere traurig sind.
36. Es fällt mir leicht zu verstehen, warum Andere sich ekeln, wenn ihnen etwas Ekelerregendes passiert.
37. Wenn mir ein Freund/eine Freundin von etwas erzählt, das ihn bzw. sie ärgert, ärgere ich mich auch leicht.
38. Ich werde leicht ärgerlich, wenn Andere sich ärgern.
39. Wenn mir ein Freund/eine Freundin von etwas erzählt, das ihn bzw. sie überrascht, kann ich leicht die Überraschung mitfühlen.
40. Es fällt mir schwer, zu verstehen, was Andere freut.
41. Ich freue mich, wenn einer fremden Person etwas passiert, was ihn/sie freut.
42. Es fällt mir schwer, vorauszusehen, welche Situationen Andere traurig machen.
43. Wenn mir ein Freund/eine Freundin von etwas erzählt, das ihn bzw. sie traurig macht, werde ich auch leicht traurig.
44. Wenn mir ein Freund/eine Freundin von etwas erzählt, das ihn bzw.
45. sie ängstigt macht, ängstige ich mich auch leicht.
46. Es fällt mir schwer, vorauszusehen, welche Situationen Andere erfreuen.
47. Wenn jemand mir von etwas erzählt, das ihn bzw. sie freut, kann ich leicht verstehen, warum es ihn/sie freut.
48. Wenn ich sehe, dass ein Freund traurig ist, bin ich auch schnell traurig.
49. Es fällt mir schwer vorauszusehen, welche Situationen Andere ekeln.
50. Es fällt mir schwer, zu verstehen, was meine Freunde überrascht.
51. Es fällt mir schwer, zu verstehen, was meine Freunde ärgert.
52. Es fällt mir schwer, zu verstehen, was meine Freunde traurig macht.
53. Ich bin auch überrascht, wenn ich sehe, dass einer fremden Person etwas passiert, was ihn/sie überrascht.
54. Ich werde traurig, wenn ich sehe, dass einer fremden Person etwas passiert, was ihn/sie traurig macht.
55. Ich kann es leicht nachvollziehen, warum Andere ärgerlich werden, wenn ihnen etwas Unangenehmes passiert.
56. Wenn ich sehe, dass ein Freund von mir, sich wegen etwas ängstigt, kann ich mich auch leicht ängstigen.
57. Ich kann es leicht nachvollziehen, wenn meine Freunde überrascht sind.
58. Wenn jemand mir von etwas erzählt, das ihn bzw. sie traurig macht, kann ich leicht verstehen, warum es ihn/sie traurig macht.
59. Ich ekele mich leicht, wenn Andere sich ekeln.
60. Wenn jemand mir von etwas erzählt, das ihn bzw. sie ängstigt, kann ich leicht verstehen, warum es ihn/sie ängstigt.
